# Spike on a bike: mobile harm reduction in action

**DOI:** 10.1186/s12954-025-01394-7

**Published:** 2026-03-25

**Authors:** Joseph Janes, Siân Roberts, Gareth Morgan

**Affiliations:** 1 Swansea University, Swansea, Wales, UK; 2 BAROD, Swansea, Wales, UK; 3 Hywel Dda Health Board, Swansea, Wales, UK

## Abstract

**Background:**

Access to harm reduction services remains a significant challenge in rural areas, where geographic and structural barriers can prevent people who use drugs from engaging with fixed-site support. In response to declining service use during the COVID-19 pandemic, the Dyfed Drugs and Alcohol Service (DDAS), led by Barod, a Welsh harm reduction and treatment provider, developed a peer-led mobile intervention known as *Spike on a Bike* (SOAB), designed to deliver harm reduction equipment and relational support directly to service users in remote communities.

**Methods:**

This study draws on a mixed-methods evaluation of SOAB conducted between May 2022 and June 2024. Quantitative data on service uptake and demographics were analysed alongside qualitative data from semi-structured interviews, online surveys, and field observations. The evaluation was designed collaboratively with partners from Swansea University’s Global Drug Policy Observatory, Public Health Wales, and local health boards.

**Results:**

SOAB significantly improved access to harm reduction support in rural West Wales, reaching individuals across diverse age groups and engaging a higher-than-typical proportion of female clients (36%). Service users reported positive experiences, particularly regarding ease of access and reduced stigma. However, uptake of blood-borne virus (BBV) testing and steroid harm reduction kits was low, indicating areas for targeted service development.

**Conclusion:**

Mobile harm reduction services like SOAB offer a promising model for addressing service exclusion in rural areas. By tailoring delivery to local contexts and reducing barriers linked to transport, stigma, and service visibility, such models have the potential to reach populations underserved by conventional approaches and inform future harm reduction strategies grounded in community knowledge and context-specific practice.

## Background

Mobile harm reduction services have emerged as a critical response to structural inequalities in healthcare access for people who use drugs, particularly in rural, remote, and underserved areas. These populations often face compounded barriers, including geographic isolation, poverty, stigma, and lack of transport, that restrict their access to traditional, fixed-site services [12, 39]. As such, the decentralisation of harm reduction through outreach or mobile provision offers a more accessible, low-threshold approach to service engagement. Across diverse contexts internationally, such interventions have been used to deliver sterile injecting equipment, naloxone, harm reduction information, and broader health advice directly to communities that may otherwise be excluded from care [19, 52].

These mobile interventions can be understood through the lens of structural vulnerability [46], which highlights how overlapping social, economic, and political structures constrain the health and autonomy of marginalised populations. For people who use drugs in rural settings, vulnerability is shaped not only by drug policy and criminalisation but also by systemic conditions such as poor transport infrastructure, digital exclusion, limited availability of local services, and heightened stigma often associated with close-knit rural communities. In this context, mobile harm reduction represents a pragmatic and adaptive response to these layered structural barriers, delivering care in ways that extend beyond the reach of conventional, facility-based models. Innovative approaches such as Canada’s mobile overdose response services (MORS) and mobile supervised consumption initiatives have demonstrated the potential of such models to address critical service gaps in rural and remote areas [39, 52].

This study is also guided by principles of low-threshold and person-centred care [41], which prioritise harm reduction, dignity, and accessibility over abstinence-based or punitive approaches. These principles are particularly important in rural settings where relationships of trust can be key to sustained engagement, and where formal services may operate with limited capacity or inflexible entry requirements. By embedding these values within a mobile framework, initiatives like *Spike on a Bike* (SOAB) offer an alternative vision of care, one that seeks to meet people where they are, both geographically and socially.

The COVID-19 pandemic exacerbated existing service inequalities and exposed the fragility of harm reduction infrastructures worldwide. Lockdowns and public health restrictions triggered widespread disruptions in drug service delivery, disproportionately affecting people who use drugs in rural areas [6, 31, 51, 61]. In Dyfed, a predominantly rural region in West Wales, the Dyfed Drug and Alcohol Service (DDAS) observed a marked decline in in-person service uptake during this period, underscoring both the centrality and precarity of harm reduction delivery in such areas.

In response, DDAS co-developed an innovative, peer-informed programme called *Spike on a Bike* (SOAB). This mobile outreach service involves trained staff delivering pre-ordered harm reduction equipment, including injecting paraphernalia and naloxone, by motorbike to clients across dispersed rural communities. Initially framed as a crisis response to lockdown conditions, the service was quickly recognised as a viable long-term intervention, co-designed by practitioners, public health officials, and frontline workers to create a more relational, flexible, and user-informed model of rural harm reduction. By combining digital ordering systems with in-person delivery, SOAB seeks to balance accessibility, discretion, and safety while also building rapport and trust among clients who are often disengaged from mainstream drug services.

Taken together, these studies point to both the potential and the under-explored nature of mobile harm reduction in rural UK contexts. This model builds on similar international initiatives, such as Vancouver’s peer-led syringe recovery initiatives and mobile naloxone delivery services in the United States [26, 34] but remains rare in a UK context and entirely novel in rural Wales.

To contextualise the evaluation and foreground its contribution, this section reviews relevant literature on harm reduction access, mobile outreach models, and rural-specific challenges. It situates the Spike on a Bike intervention within broader debates around innovation, equity, and structural harm as they relate to geography and health access. This study, therefore, evaluates Spike on a Bike (SOAB), situating it within existing evidence on rural harm reduction.

## Literature review

To contextualise the evaluation of SOAB, this review considers existing scholarship on harm reduction access, rural exclusion, and mobile and peer-led outreach. It begins by outlining evidence on harm reduction and service access, before examining how rurality and structural inequalities create distinctive barriers. It then turns to international literature on mobile and digital outreach models, highlighting their potential relevance for the Welsh context.

### Harm reduction and access to services

Harm reduction interventions, including needle and syringe programmes (NSPs), opioid agonist therapy (OAT), take-home naloxone distribution, and blood-borne virus (BBV) screening, are widely recognised as effective measures for reducing drug-related harms, especially those linked to infectious disease transmission and overdose [2, 36]. However, the impact of these interventions is often constrained by persistent barriers to access. Structural determinants such as poverty, criminalisation, stigma, and inadequate housing continue to deter engagement [47]. Crucially, these barriers are unevenly distributed, with those at highest risk frequently the least able to access support [62]. In particular, people who use drugs in non-urban environments remain largely excluded from mainstream service design, policy discourse, and empirical study. Such exclusions generate not only individual-level risks but also entrench systemic health inequalities at a broader population level [13].

### Geographies of exclusion: rurality and structural harm

The spatial configuration of public health infrastructure plays a key role in shaping access to harm reduction. In the UK, services are predominantly concentrated in urban areas, informed by population density, funding allocations, and institutional assumptions about drug use patterns [63]. This has led to the formation of what [21] refer to as “geographies of exclusion,” where individuals in rural and semi-rural areas encounter multiple, intersecting barriers to engagement. These include long travel distances, insufficient public transport, digital exclusion, and heightened community visibility, all of which constrain health-seeking behaviours [53]. In the Welsh context, where over one-third of the population resides outside of urban centres [65, 66], these spatial inequalities are especially acute. As Woods [68] notes, rural deprivation in Wales is frequently masked by idyllic representations of the countryside, which can obscure the socio-economic realities of those living in rural poverty. Similarly, Gibbons et al. [20] argue that rural Welsh communities experience structural harm in layered ways, from housing precarity to the erosion of public services and out-migration of young people. These overlapping factors limit both the availability of harm reduction services and the capacity or willingness of rural populations to access them.

### Mobile harm reduction: reaching the unreachable

Mobile harm reduction models have emerged as pragmatic responses to the limitations of static, site-based services. These models provide enhanced adaptability, discretion, and reach, enabling services to connect with people who may be geographically or socially distant from conventional provision [58, 59]. Evidence from international settings, including mobile supervised injection units in Vancouver and mobile BBV screening in Australia, demonstrates that mobile outreach can build trust, reduce stigma, and support increased service uptake among people considered disengaged from mainstream systems [33, 67]. Their temporal flexibility, operating outside traditional office hours or in locations aligned with user routines, further enhances their accessibility [44]. Despite the demonstrated efficacy of these models, there remains a notable gap in their implementation and evaluation within rural settings. In the UK, the literature on mobile harm reduction has predominantly focused on urban street-based provision or festival-based drug checking [38]. Given the distinct policy landscape and geographical configuration of rural Wales, shaped by devolved governance, austerity, and infrastructural challenges, there is a critical need to examine how mobile harm reduction can be adapted to and sustained in such contexts.

### Digital and peer-led outreach

Complementing mobile models, harm reduction innovations increasingly utilise digital technologies and peer-led approaches to widen engagement. Discreet QR codes, geo-located harm reduction apps, and social media platforms now serve as low-threshold entry points for accessing information and support, particularly for younger, tech-literate populations [8]. These tools are especially valuable in environments where stigma or surveillance discourages in-person contact [40]. Simultaneously, peer-led models, where individuals with lived experience of substance use play central roles in service delivery, have proven effective in building trust, increasing cultural relevance, and improving access [3, 28]. Peer workers often possess deep knowledge of the “hidden scripts” of drug-using communities, allowing them to navigate informal networks and support engagement in ways traditional professionals may not [24]. Although the integration of digital and peer-led strategies has shown promise in urban settings, their application in rural harm reduction remains under-examined. Yet, emerging research suggests that digital inclusion can act as a pathway through which service users access support more safely and effectively, particularly in marginalised settings [32, 56].

### Addressing the gap: a welsh model of rural harm reduction

Despite international advancements in mobile, digital, and peer-informed harm reduction, the UK evidence base, particularly within devolved, rural settings, remains limited. Wales, with its devolved public health system and significant rural population, presents a distinctive context for exploring harm reduction innovation (NHS Confederation 2025) [42]. However, scholarly attention has largely concentrated on urban policy developments, overlooking how harm reduction operates at the intersection of rurality, marginalisation, and health inequality. This paper addresses that gap through an in-depth evaluation of the Spike on a Bike (SOAB) initiative, a mobile harm reduction programme developed in West Wales by a partnership of public health and third-sector actors. In doing so, the study contributes to the growing body of literature on structural harm and service accessibility, offering practical insights into how relational, adaptive, and context-sensitive interventions can expand the boundaries of care in underserved geographies.

Despite international evidence on mobile harm reduction, there remains little empirical research from rural UK contexts. This study therefore evaluates Spike on a Bike (SOAB), a peer-led mobile harm reduction programme developed in rural West Wales in response to COVID-19 service disruptions. The following section provides a brief overview of the programme.

## The spike on a bike programme: a mobile harm reduction response in rural Wales

In Wales, these challenges prompted the development of Spike on a Bike (SOAB), a mobile harm reduction initiative designed to address service disruption during the COVID-19 pandemic. While described in detail earlier (see Background), here we situate SOAB within broader international developments, drawing on evidence from mobile supervised consumption services in Canada [33], mobile BBV testing in Australia [67], and other outreach models that inform its design. What remains underexplored, however, is how such an approach functions in the distinctive Welsh rural context.

**Fig. 1:**
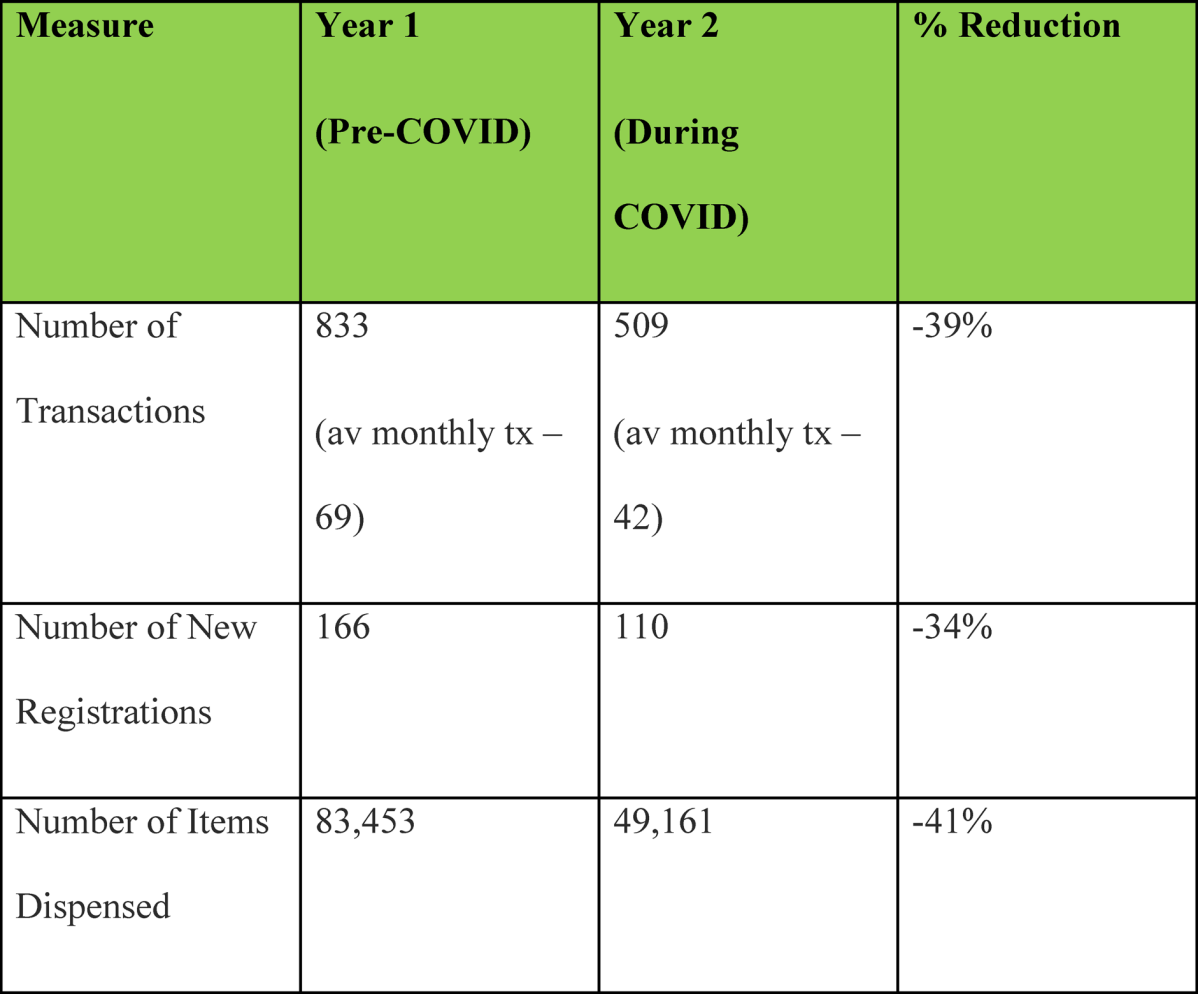
DDAS using Llanelli and Aber NEX data for Dyfed. (Pre COVID and during COVID statistics)

In Dyfed, a notable decline in engagement with fixed-site services during the pandemic (see Fig. 1) catalysed the creation of SOAB as a more flexible and resilient approach to maintaining harm reduction provision.

The programme’s co-design with local stakeholders, service providers, health professionals, and third-sector partners was intended to respond to local needs.

SOAB specifically targeted rural communities that have historically experienced exclusion from fixed-site services due to distance, stigma, and digital inaccessibility [31, 63]. By offering doorstep delivery, the initiative improved service reach and client safety while also providing relational continuity through repeat engagement and trust-building. The integration of digital technologies, including discreet online ordering systems and service portals, further enhanced accessibility and supported the anonymity required in highly surveilled rural settings.

SOAB represents a hybrid model that fuses mobile and digital outreach with principles of low-threshold, person-centred care. Although such innovations are increasingly discussed in the international literature, their implementation in rural UK settings remains nascent. This evaluation contributes to addressing that gap by assessing the design, delivery, and impact of SOAB within the specific public health landscape of Wales. Drawing on qualitative and quantitative data, the study examines how the programme navigates structural exclusion and adapts to the complex realities of rural harm reduction delivery. In light of these gaps, this study evaluates the Spike on a Bike initiative with two aims: (1) to examine how mobile harm reduction operates within the rural Welsh context; and (2) to assess its potential for sustainability and integration within wider harm reduction systems. The following section outlines the methodological approach employed to evaluate the SOAB intervention and its implications for future practice.

## Methods

### Study design

This study presents an exploratory, mixed-methods evaluation of *Spike on a Bike* (SOAB), a mobile harm reduction initiative operating across rural areas of Wales. Conducted by researchers from the Global Drug Policy Observatory (GDPO) at Swansea University between May 2022 and June 2023, the evaluation aimed to assess the implementation and delivery of the SOAB programme from the perspectives of both service providers and people who use drugs. The goal was to identify core strengths, limitations, and opportunities for development in rural harm reduction practice.

A mixed-methods approach was adopted in alignment with the study’s interpretivist epistemology, which emphasises contextualised, experiential knowledge over generalisable claims. Quantitative service data (e.g. order metrics) offered a descriptive overview of activity, while qualitative interviews, online surveys, and ethnographic observations enabled a deeper exploration of how participants experienced the programme. This integration supported a relational, meaning-centred inquiry, allowing the evaluation to surface both operational dynamics and the lived realities of harm reduction in rural settings.

### Epistemological framework

The study was grounded in an interpretivist epistemology, which holds that knowledge is not objective or fixed, but constructed through social interaction, language, and context [22, 50]. Within this paradigm, lived experience and relational meaning-making are prioritised over predictive generalisation. This approach was particularly well suited to engaging with structurally marginalised populations, where official narratives often obscure the realities of exclusion, harm, and resilience.

Data collection was situated within a rapid ethnographic framework [1, 64]. Rapid ethnography is increasingly used in health and public service research to generate timely, context-rich insight without the extended immersion of traditional ethnography. While shorter in duration, it maintains a commitment to situated, relational understanding. Within this framework, participant observation was used not simply to observe, but to engage in real-world settings, building trust, witnessing informal peer dynamics, and capturing everyday interactions that often go unspoken in formal interviews [10, 15]. These insights helped surface the hidden labour of peer workers, including their emotional and advocacy roles, and revealed how geographic and affective contexts shaped access to care [30].

### Sampling

A multi-method strategy was adopted, combining semi-structured interviews, participant observation, and a targeted online survey. Participants were identified through purposive sampling in collaboration with Dyfed Drug and Alcohol Service (DDAS), with practitioners and outreach staff introducing clients who had interacted with SOAB and expressed willingness to participate.

In total, the study involved eight semi-structured interviews with practitioners, policy stakeholders, and project leads; five semi-structured interviews with service users; and ten service users who completed an online survey comprising a mix of closed and open-ended items.

Interview questions were guided by a topic framework that included themes such as access, stigma, outreach visibility, service trust, and perceived benefits. These themes were drawn from existing harm reduction literature and refined in consultation with project stakeholders. The interview format was consistent across groups, although flexibility was maintained to allow for narrative depth and contextual sensitivity. The survey aimed to complement the interview data by capturing broader perspectives, particularly from individuals less comfortable with formal participation. Survey participants were recruited via outreach staff and flyers shared during SOAB visits. Political and ethical constraints prevented broader public advertising.

### Data collection

Data collection was conducted between May 2022 and June 2023. Over this period, the research team undertook twelve outreach sessions alongside the SOAB team, as well as several multi-agency meetings involving DDAS and Health Board staff. In-depth fieldnotes were recorded immediately after each engagement, documenting informal interactions, spatial dynamics, and logistical challenges encountered in the field. In addition, all interviews were audio-recorded and transcribed verbatim, while online survey responses were anonymised and securely stored for analysis.

The lead author was embedded within the project team through their role at the Global Drug Policy Observatory. This proximity enabled trusted access and insider-supported recruitment [7]. At the same time, it required active reflexivity to manage dual roles as researcher and collaborator, to avoid blurring advocacy with analysis [14, 37].

Participant observation was conducted by the lead researcher, who accompanied SOAB staff on twelve outreach sessions between May 2022 and June 2023. The researcher observed service delivery in real-time and engaged informally with both staff and service users. Observations focused on interactions, trust-building processes, and logistical challenges of delivering mobile harm reduction in rural areas. Detailed field notes were written immediately after each session.

The online survey combined closed questions (e.g., demographics, frequency of SOAB use) with open-ended free-text items aligned to the interview guide. These open-ended questions invited participants to comment on access, stigma, and perceived benefits of SOAB. While shorter than the interviews, they provided qualitative insight from individuals who preferred not to take part in longer conversations.

### Data analysis

Data were analysed thematically using Braun and Clarke’s [9] six-step approach. NVivo software supported data organisation and coding. Initial codes were generated inductively and refined into broader themes through team discussion. A reflexive approach was maintained, with particular attention paid to how the researcher’s positionality and embeddedness shaped interpretation. Findings were triangulated across interviews, survey responses, and observations to strengthen credibility.

Service use data, including order metrics and demographic breakdowns, were collected and analysed to provide context for the qualitative findings. These metrics were not used to generate causal claims but to help visualise the scale and diversity of engagement.

Findings were summarised using a strengths and weaknesses matrix aligned with developmental evaluation principles [43], identifying both areas of good practice and opportunities for system-level improvement.

### Ethics

Ethical approval was granted by the School of Social Sciences Ethics Sub-Committee at Swansea University. The evaluation followed the British Society of Criminology Code of Ethics (2015) and Swansea University’s research governance protocols. Written informed consent was obtained from all participants. They were assured of confidentiality and their right to withdraw at any point; no participants chose to do so. Interview settings were selected based on participant comfort, and staff were trained to ensure a trauma-informed and non-coercive approach throughout.

## Results

The following section presents the evaluation findings, structured thematically to reflect both programme-level outcomes and specific areas of good practice and development. Findings are derived from interviews and survey data, observational insights, and service use metrics. The results highlight the Spike on a Bike (SOAB) programme's role in addressing gaps in rural harm reduction, its capacity to engage underrepresented populations, and the barriers that limit optimal impact.

### Expanding harm reduction access in rural areas

SOAB demonstrated significant success in delivering harm reduction services to people in rural and semi-rural communities who face multiple access barriers, including geographic isolation, poor public transport, and digital exclusion. Practitioners emphasised the value of a discrete, flexible, and mobile approach, which enabled consistent and proactive contact with service users. This relational model allowed workers to build trust incrementally and tailor support to the unique needs of individuals over time.

*"We’re getting to people no one else is reaching, isolated communities, individuals living far from town centres, and those not engaging with fixed sites."* (Rider, SOAB).

Such contact was particularly valuable in engaging individuals previously disconnected from formal health or social care services.

### Expanding access among underserved groups

A notable feature of SOAB’s reach was its ability to engage populations typically underserved by harm reduction. Thirty-six percent of orders were placed by female clients, a proportion notably higher than typically reported in fixed-site NSP services. The age diversity of clients ranged from under-20s to over-50s. Practitioners noted that SOAB’s informal, user-led design helped reduce anxiety around accessing services, especially for those who had not previously engaged with harm reduction provision.

### Equipment distribution and harm reduction supplies

Between May 2022 and June 2023, *Spike on a Bike* (SOAB) distributed 141 naloxone kits (Prenoxad and Nyxoid) through mobile outreach, directly contributing to local overdose prevention efforts. Alongside formal orders placed online, SOAB supports peer-to-peer naloxone distribution through trusted networks [17]. This model enables the reach of individuals who may not engage with fixed-site services and supports decentralised overdose response efforts [18]. In collaboration with Dyfed Drug and Alcohol Service (DDAS), SOAB implemented a peer-distribution model, training individuals with lived experience to disseminate naloxone within their communities. This peer-led strategy contributed to at least two confirmed overdose reversals.

*"If the rider had not engaged with me several times, I wouldn’t have come into the service."* (Service user testimony).

**Fig. 2:**
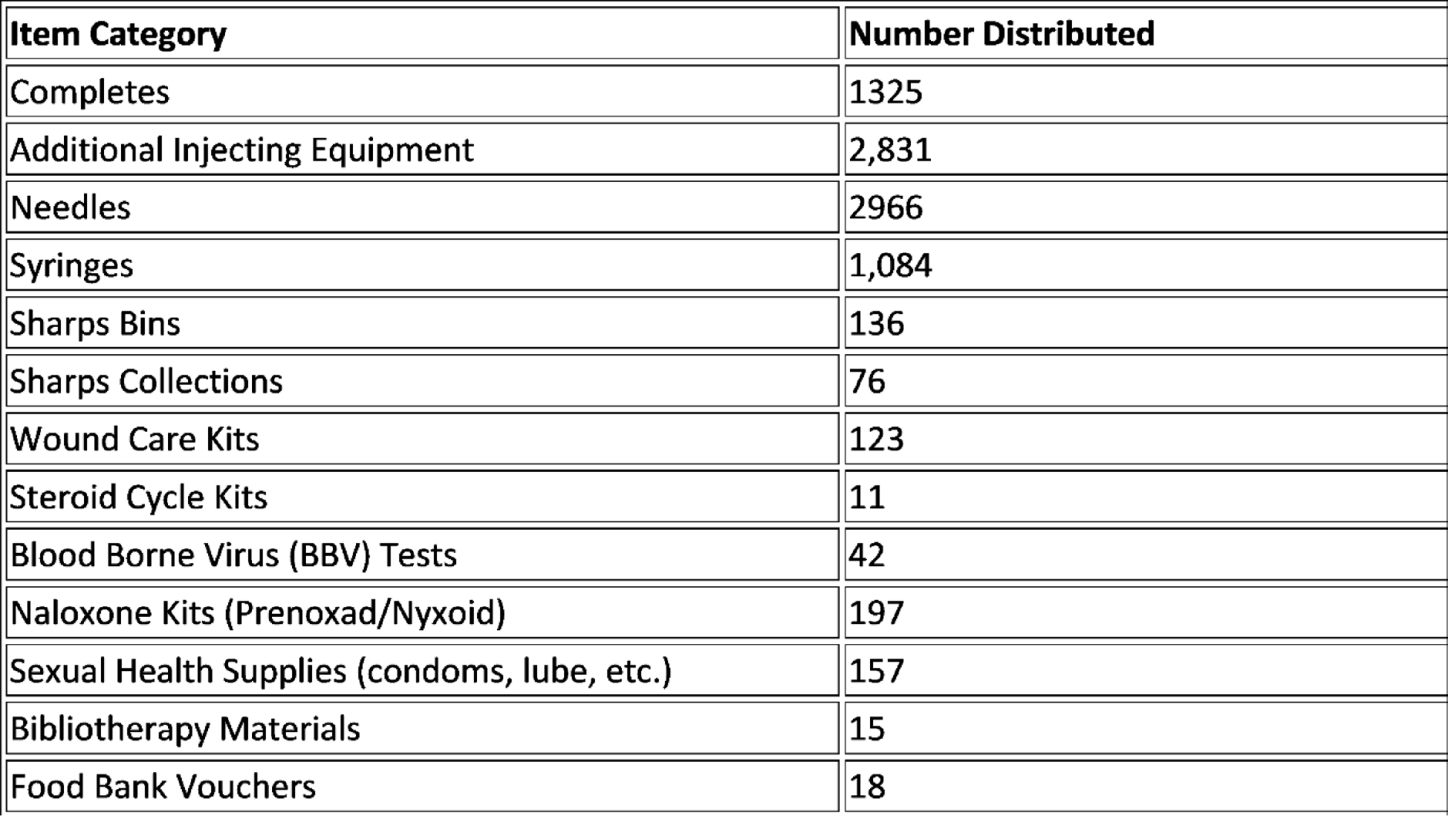
Summary of Harm Reduction and Support Supplies Distributed via SOAB (May 2022–June 2023). Note: Data represents recorded transactions via SOAB’s mobile outreach platform

In addition to naloxone, SOAB supplies a wide range of injecting and sexual health equipment via both pre-order and spontaneous distribution. Outreach workers carry a mobile reserve of sterile syringes, dry bloodspot testing kits, needles, foil, sharps bins, condoms, and lubricant. This allows them to respond flexibly to community needs and support initial engagement with individuals not yet formally registered. New clients are typically reached via peer outreach, QR code flyers, or word-of-mouth referrals. A summary of key service delivery metrics from May 2022 to June 2023 is presented in Fig. 2 to illustrate the programme’s reach and scope.

These distribution patterns reflect the programme’s emphasis on practical, person-centred care, with equipment tailored to individual needs rather than one-size-fits-all interventions. The inclusion of items such as food bank vouchers, wound care kits, and bibliotherapy resources also highlights SOAB’s responsiveness to wider social and emotional needs, reinforcing the model’s holistic ethos. Quantifying this activity helps demonstrate the breadth and flexibility of outreach work, often obscured in purely qualitative accounts.

These figures highlight the breadth of support provided beyond traditional injecting equipment, reinforcing SOAB’s commitment to holistic harm reduction and community wellbeing.

While harm reduction coverage in the UK has increased in many areas, legal restrictions under the Misuse of Drugs Act currently prohibit the distribution of safer inhalation devices (SIDs) such as crack pipes.

### Barriers to BBV testing uptake

Despite strong engagement with equipment and naloxone distribution, BBV testing uptake was comparatively low. Only 0.5% of all orders included dried blood spot testing, in contrast to 33% of orders for needles and 32% for other paraphernalia. Practitioners cited uncertainty around test visibility on the ordering platform and concerns from clients about privacy or follow-up contact. Stakeholders cited concerns about digital design, privacy, and awareness.

### Service visibility and political constraints

SOAB’s implementation was challenged by limited political and community support in certain areas, particularly Llanelli. Local resistance to a proposed fixed-site hub and concerns about reputational damage curtailed promotion efforts and undermined service visibility. Practitioners identified these tensions as key obstacles to embedding the programme more widely.

### Stigma, trust, and holistic harm reduction

Clients repeatedly emphasised the significance of SOAB’s informal, community-embedded model in helping them overcome the layered stigma surrounding drug use. Several participants described the mobile outreach team as “non-judgmental,” “respectful,” and “safe.” Beyond core harm reduction supplies, SOAB also distributed condoms, sanitary products, and food bank vouchers. These items, while not traditionally included in drug service provision, recognise the social determinants of health and frame harm reduction as part of a broader wellbeing agenda.

SOAB’s agility also enabled responsive service design and early intervention. Inspired in part by Canada’s Spikes on Bikes model [55], staff could adjust routes in real time and respond rapidly to emerging needs, including individuals not yet in treatment. Taken together, these findings underscore SOAB’s ability to deliver flexible, relational, and context-specific care. The following discussion considers these findings in light of broader harm reduction scholarship and ongoing policy challenges.

## Discussion

The article and subsequent discussion contribute to the growing international literature on mobile and outreach-based harm reduction and offer context-specific insights into the innovations required to engage hidden or underserved populations, particularly in settings where structural exclusion is intensified by rurality, austerity, and the centralisation of services. SOAB’s operational success affirms international evidence that mobile outreach is essential in contexts where fixed-site services are inaccessible, stigmatised, or distrusted [39, 44].

### Reducing structural barriers in rural harm reduction

SOAB’s mobility and informality enabled access for individuals facing compounded exclusion due to geography, transport, and digital literacy. The service provided not only sterile injecting equipment but also a means of re-entering broader health systems. As one participant described, *"I would never have engaged with services if it was not for Spike on a Bike,"* reflecting a key insight: accessibility is not only spatial but relational and emotional, echoing international findings on the efficacy of mobile outreach in fostering sustained engagement [60].

The relational approaches to foster relationships helped to mitigate structural, social, and internalised (self) stigma, barriers that frequently prevent people from engaging with fixed-site services. This approach aligns with harm reduction literature that stresses non-coercive, person-centred care as central to service effectiveness [48].

Responding to secondary service user needs was seen as a strength. The inclusion of wider non-traditional service provision (condoms, sanitary products, and food bank vouchers), which recognised the social determinants of health and framed harm reduction as part of a broader wellbeing agenda, helped establish rapport and positioned SOAB as a supportive, community-centred intervention rather than a surveillance-oriented service. This approach reflects a growing shift toward integrated care in harm reduction [29].

### Engagement with women and young people

SOAB’s higher engagement from female service users suggests that the model helped address gender-specific barriers that typically limit women’s access to harm reduction, such as surveillance, stigma, and fear of child removal [45]. Mobile, non-institutional contact allowed for more informal trust-building, and peer-led engagement may have made the service appear safer and less judgmental than fixed-site alternatives. Similarly, young people may prefer informal, non-institutional interactions, suggesting that flexible models like SOAB are particularly well-suited to reaching early-stage or hidden users. This reinforces the importance of designing gender-responsive outreach models.

### Addressing BBV testing gaps

The low uptake of BBV testing, despite strong engagement with other harm reduction supplies, highlights a key shortfall in the model’s integrated care potential. This represents a missed opportunity in advancing Wales’s public health goals, including the elimination of hepatitis B and C by 2030. Barriers may include digital friction [4], lack of test visibility, and service users’ uncertainty about follow-up. These barriers not only reflect interface design challenges but also shape users' sense of safety, privacy, and willingness to disclose. This aligns with broader critiques that even progressive harm reduction services can underdeliver on diagnostics when those are not embedded into user-friendly pathways [58]. Evidence suggests that poor user experience, especially requiring more than three user actions, can significantly reduce engagement [4, 27]. Repositioning BBV testing options earlier in the digital journey may improve uptake.

### Peer-led interventions as a strength

SOAB’s success in implementing peer-to-peer naloxone distribution underlines the critical role of lived experience in public health delivery. Peers acted not only as distributors but as connectors, increasing trust and accessibility. This aligns with wider calls for formal recognition and compensation of peer workers within harm reduction systems [16]. SOAB reflects a wider evidence base demonstrating the effectiveness of peer-led harm reduction. International research highlights that peer naloxone delivery enhances uptake, fosters resilience within drug-using communities, and challenges the marginalisation of lived experience within health systems [35].

### Navigating political and cultural resistance

The service’s limitations in Llanelli underscore a persistent challenge: the contested nature of harm reduction in local politics. Moral stigma and misconceptions about enabling drug use remain powerful barriers to expansion [47]. Local councillors expressed concerns consistent with what is often described as *'Not in My Back Yard'*, where residents oppose the siting of services in their immediate area despite acknowledging the broader need. Future scale-up will depend on stronger community engagement strategies, political advocacy, and the normalisation of harm reduction as a legitimate, evidence-based public health response.

A further navigation pertains to the legal restrictions under the Misuse of Drugs Act, which prohibit the distribution of safer inhalation devices (SIDs) such as crack pipes. This limitation persists despite emerging evidence on the public health benefits of such interventions. An estimated 180,000 people use crack cocaine in England [25], yet many rely on homemade pipes or reuse shared equipment, elevating the risk of viral transmission and respiratory injury.

International evidence shows that distributing crack pipes can reduce equipment sharing, related injuries, and transitions to injecting, while improving service uptake. In the UK, the NIHR-funded Safe Inhalation Pipe Provision (SIPP) study, the first of its kind, aims to evaluate this approach and inform future harm reduction policy [25]. In Wales, [5] policy briefing similarly highlights reduced paraphernalia sharing and health risks when safer inhalation devices (SIDs) are informally accessed. Although SOAB cannot distribute SIDs due to legal restrictions, peer teams continue to gather service user insights, track policy shifts, and support advocacy for evidence-based reform.

### Broadening the concept of harm reduction

SOAB’s distribution of food, sanitary products, and sexual health supplies signals a meaningful shift in the scope of harm reduction, from a traditionally biomedical focus centred on disease prevention and overdose reversal, toward a more holistic, socially grounded model of care. This broader framing positions harm reduction as not merely a response to individual health risks but as a platform for addressing the intersecting social determinants of health, including poverty, housing instability, and gendered disadvantage.

Such an approach is increasingly advocated within harm reduction literature, where calls to move “beyond the needle” have challenged the sector to engage with wider forms of structural vulnerability [3]. Jozaghi and Bird [29], for example, argue that meaningful harm reduction must address both immediate risks (e.g., infectious disease or overdose) and the underlying conditions that shape them, including food insecurity, stigma, and systemic marginalisation. SOAB’s delivery of essential non-medical items reflects this evolving ethos, with services tailored not only to reduce harm but to promote dignity, autonomy, and inclusion.

Crucially, this expanded model aligns with the lived realities of many service users, whose needs are not siloed but shaped by overlapping forms of social and economic exclusion. By responding to these needs in a flexible, low-threshold way, SOAB demonstrates how mobile harm reduction can act as a gateway to broader forms of care and support, challenging assumptions that these services should be narrowly clinical and instead reframing them as crucial components of social justice and public health infrastructure. The flexibility of service delivery echoed this and was especially critical in rural areas, where structural barriers often delay or prevent timely access to harm reduction services [49].

### Policy and scalability implications

The findings from SOAB offer actionable insights for policymakers and public health planners aiming to improve harm reduction delivery in rural settings. Embedding flexibility and mobility into service design is critical to overcoming the geographic and infrastructural barriers that often limit access in dispersed and underserved communities. The effectiveness of peer-led approaches, as demonstrated in this project, highlights the importance of providing formal training, sustained support, and fair remuneration for people with lived experience who play key roles in outreach and engagement.

These challenges reflect broader patterns documented in harm reduction literature, where moral panic, stigma, community opposition, and policy inertia constrain service development [11, 47, 54]. Overcoming such barriers requires sustained political advocacy and stakeholder education to foster supportive environments for innovative, evidence-based interventions [57], [23].

Improving access to ancillary services such as blood-borne virus (BBV) testing also requires better integration and streamlining of digital interfaces to reduce friction and enhance usability, particularly for marginalised populations. In addition, efforts to scale harm reduction initiatives must prioritise community education and stakeholder engagement to build political legitimacy and counteract stigma, both of which can act as hidden barriers to service uptake and sustainability.

Ultimately, positioning harm reduction within a broader social care and public health framework, one that addresses not only individual drug use but also the social determinants of wellbeing, is essential. Scaling up programmes like SOAB requires not only operational investment but also enabling policy environments that foster innovation, promote cross-sector collaboration, and support the meaningful involvement of people with lived experience in service design, delivery, and evaluation.

## Conclusion

The Spike on a Bike (SOAB) programme offers a promising model that warrants further study of its transferability for low-threshold, mobile harm reduction delivery in areas marked by rurality, structural exclusion, and service disengagement. Its success in engaging women, younger adults, and underserved populations demonstrates the effectiveness of trust-based, peer-informed, and discreet interventions tailored to local needs. More than a logistical delivery mechanism, SOAB is rooted in community insight and shaped by the lived experiences of those it seeks to support.

While the programme has delivered critical benefits, particularly in naloxone distribution and rural outreach, opportunities remain to strengthen its digital accessibility, increase BBV testing uptake, and deepen relational ties within local health systems.

The evaluation of *Spike on a Bike* demonstrates its ability to reach underserved populations and deliver harm reduction in a relational, person-centred manner, while also highlighting ongoing limitations, such as low uptake of BBV testing. Future research could examine the service’s transferability to other contexts, potential for scaling, and opportunities to enhance digital integration and user engagement. As a mobile, adaptive model, *Spike on a Bike* offers valuable insights into how harm reduction initiatives can navigate logistical, social, and institutional challenges, providing evidence to inform both local practice and broader harm reduction strategies in similarly underserved settings.

## Data Availability

J.J. owns the data set, access to the data on request through J.J. and DDAS.
